# Seasonal Variation of Gut Bacterial Community in Wild *Apodemus agrarius* Is Associated With Intrinsic and Extrinsic Environments

**DOI:** 10.1002/ece3.74257

**Published:** 2026-09-01

**Authors:** Yizhen Zhang, Yan Chen, Zhijiang Bai, Xinliang Zhang, Deng Wang

**Affiliations:** ^1^ College of Grassland Science and Technology China Agricultural University Beijing People's Republic of China; ^2^ Yuqing Plant Protection and Quarantine Station Zunyi City Guizhou Province People's Republic of China; ^3^ Yunnan Provincial Key Laboratory of Animal Nutrition and Feed, Department of Pratacultural Science, Faculty of Animal Science and Technology Yunnan Agricultural University Kunming People's Republic of China

**Keywords:** *Apodemus agrarius*, body size, environmental factor, gut bacteria, reproductive intensity, seasonality

## Abstract

Environmental factors and the reproductive status of animals have been reported to influence host gut microbial community structure significantly. To date, there is a lack of integrated research reporting the relationships among environmental factors, gut microbial structure, and the life processes in animals. This study collected monthly temperature, precipitation, and vegetation indices in the sampling site, and body size metrics as well as reproductive indicators in wild *Apodemus agrarius* (Pallas, 1771) from 2016 to 2023; and also collected gut microbes in the monthly animal populations from March to December in 2023, and analyzed the relationships among climatic factors, gut microbiota structural features, body size, and reproductive indicators in the animal population. The results showed that both variations of monthly reproductive intensity and the α‐diversity of gut bacterial community in *A. agrarius* population followed an “M”‐shaped pattern over months. The mean monthly temperature, total precipitation, and mean monthly NDVI were significantly associated with α‐diversity, reproductive intensity, and body size. The exploratory structural equation models suggested sex‐specific associations among reproductive activity, gut bacterial alpha diversity, environmental factors, and host body size. The findings indicate correlated seasonal variation among environmental factors, gut microbial alpha diversity, and life‐history traits in rodents.

## Graphical abstract

This study reveals sex‐specific associations among seasonal reproductive activity, gut bacterial diversity, and body size in wild mice.

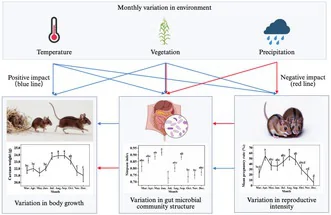

## 1 Introduction

Growth is a foundation for animal survival, reproduction, and population dynamics (Clutton‐Brock and Sheldon 2010). Animal growth involves complex processes and is influenced by a multitude of factors such as genes, nutritional availability, and extrinsic environments (Hammond 1947). Nutrition serves as the direct source of metabolic materials and available energy for the organism, providing the fundamental materials for growth. The nutrients available to animals are derived from food intake (Robbins and Cunha 2013), which is primarily digested and absorbed in gut. It has been demonstrated that gut microbiota is closely associated with essential physiological processes, including feeding, digestion, absorption, and the subsequent metabolism and transformation of nutrients (Rowland et al. 2018). Consequently, the gut microbiota was regarded as the “second genome” in a host (Zhernakova et al. 2024). A body of research has demonstrated that environmental factors significantly influence key life processes such as animal growth and reproduction (Sejian 2013), for instance, photoperiod can affect hormone secretion in *Microtus pennsylvanicus* , thereby initiating or suppressing reproductive behavior in specific seasons; temperature can directly impact energy balance, thereby affecting growth rates and reproductive performance in mammals (Bronson 2009). These effects of environmental factors on some life processes of animals (e.g., body growth) may be associated, at least in part, with gut microbiota. For instance, it has been reported that artificial rain enhancement altered plant community composition, which in turn affected the diet of *Brandt's vole*, this dietary shift was accompanied by changes in its gut microbial community structure, was subsequently linked to the production of specific metabolic products, and corresponded to the animal's growth rate (Li, Yin, et al. 2020). The growth and reproduction condition of wild animals exhibit seasonal variations (Wells et al. 2022; Henttonen and Hanski 2000), seasonal shifts in environmental factors, such as fluctuations in temperature and variations in food availability, can drive seasonal differences in life‐history traits of wild animals, such as the variation of seasonal reproductive peak, and the gut microbiota are likely associated with these processes (Carey et al. 2013; Maurice et al. 2015). Therefore, in order to establish a foundation for research on the mechanisms by which environmental factors influence animal growth, the relationships among seasonal environmental factors, gut microbial communities, and their associations with growth and reproductive traits in animals need to be clarified.

A body of literature has reported that seasonal variations in environmental factors have led to changes in plant composition of ecosystem, animal's food resource availability and trophic structure, which are known to correlate with the composition and function of the gut microbiota of animals (Li, Rui, et al. 2020; Wang et al. 2023), for instance, in overwintering animals, higher temperatures and more abundant food resources in spring and summer may promote the proliferation of dominant bacterial groups associated with nutrient absorption, while lower temperatures and food scarcity in autumn and winter likely have led to a reduction in the abundance of these microbial communities (Hao et al. 2024). Alterations in the composition and function of the gut microbiota may correspond to various biological activities in the host, such as growth rate, metabolic physiology and behavioral performance (Nguyen et al. 2021; Cryan and Dinan 2012; Turnbaugh et al. 2006), for instance, mice that received gut microbiota transplants from *Microtus montanus* exhibited enhanced tryptophan synthesis capacity and higher plasma tryptophan levels, consequently, the animals showed a dietary preference for foods with a higher protein‐to‐carbohydrate (P: C) ratio. Conversely, mice transplanted with gut microbiota from *Onychomys torridus* exhibited a stronger preference for food with a lower P: C ratio (Trevelline and Kohl 2022).

On the other hand, published literature also reported that the composition and function of gut microbiota vary with various physiological activities in a host animal, such as genetic characteristics, hormone levels, and neural regulation (Bonder et al. 2016; Wachsmuth et al. 2022; Cryan and Dinan 2012). Among these regulations, the bidirectional association between hormones and the gut microbiota is particularly evident. For instance, an increase of serum melatonin in *Lasiopodomys brandtii* leads to a rise in the abundance of Firmicutes in the animal's gut, which in turn results in a reduced secretion of gonadotropins in the body, and a subsequent shift in the peak of their circadian rhythm, the change causes a next decreased weight of the testes and ovaries, and an ultimate decline in the reproductive capacity of the animal (Zhu et al. 2022). The reproduction of small mammals exhibits distinct seasonal variation, and the energy allocation between their growth and reproduction appears to shift dynamically (McNab 2008), some studies suggest that animal may allocate more energy to foetal development through gut microbiota‐mediated metabolic pathways, thereby influencing their own growth or immune capacity (Koren et al. 2012; Rowe et al. 2020). For instance, there is a significant correlation between body size and reproductive intensity across seasons, individual growth rates tend to decrease during reproductive periods, whereas during periods of rapid growth, reproductive intensity diminished (Stearns 1998). Variation in gut microbial structure may be part of the dynamics of energy allocation between growth and reproduction in animals. However, the present studies primarily focus on the pairwise relationships among variations in environmental factors, gut microbial community structure and some life characteristics of host animals; integrated relationships among the seasonal variations of the above three variables rarely were reported.

Here, we investigated the relationships among seasonal variations in environmental factors, the gut microbiota structure, growth levels and reproductive intensity in the striped field mouse ( *Apodemus agrarius* ). This species is a small, short‐lived mammal with two generations per year, exhibiting significant seasonal variations in both individual growth levels and reproductive intensity (Yang et al. 2016; Zhang et al. 2025). We collected mean monthly temperature, total precipitation, and Normalized Difference Vegetation Index (NDVI) at the sampling site in Yuqing County, Guizhou Province; body size (body length and carcass weight) and reproductive indicators in the animal spanning March to December of each year from 2016 to 2023; and also collected gut microbes in the monthly animal populations from March to December in 2023. We analyzed (i) the seasonal patterns of temperature, precipitation, NDVI, body size and reproductive indicators of *A. agrarius* ; (ii) the seasonal variations in the gut bacterial community of *A. agrarius* ; (iii) the relationships among environmental factors, seasonal reproductive intensity, growth degree indicators and gut bacterial community structure in the animal.

## 2 Material and Methods

### 2.1 Rodent Sample Collection

We collected rodent data from 2016 to 2023 in Baini Town, Yuqing County, Guizhou Province (27.23°N, 107.90°E). Snap trapping was conducted in cornfields. Captured rodents were identified by species and sex; carcass weight and reproductive condition of individuals were recorded. Female reproductive status was determined by examining the presence or absence of embryos in the uterus and recording the number of embryos, whereas male reproductive status was assessed by checking whether the testes had descended into the scrotum. In 2023, cecal content of animals was collected and deposited in dry ice at −78°C and then transferred to a −80°C freezer for further testing. A total of 1014 adult mice (373 ♀, 641♂) were captured, of which 96 adults (37♀, 59♂) were captured in 2023 for an additional test of gut microbiota.

### 2.2 Environmental Data Collection

To assess changes in environmental variables for the study period at the sampling site, we obtained remote sensing data of 30‐m ground resolution from the Sentinel‐2 satellite via the Google Earth Engine platform and extracted mean temperature and total precipitation data from the ERA5‐land reanalysis dataset (spatial resolution approximately 11 km × 11 km, available from the European Centre for Medium‐Range Weather Forecasts). These data were used to calculate the monthly mean temperature, total precipitation, and monthly mean NDVI in 2023.

### 2.3 Gut Bacterial Sample Collection and the 16S rRNA Sequencing

We used the Ezup Genomic DNA Extraction Kit (Shanghai Sangon Biotech Co. Ltd., China) to extract DNA from approximately 0.1 g of cecal content for each mouse. According to the PCR protocol in Li et al. (2017), we amplified the V4‐V5 region of the 16S rRNA gene in the gut microbiota of each mouse using primers 515F (5′‐GTGYCAGCCGGTA‐3′) and 909R (5′‐CCCCGYCAATTCMTRAGT‐3′). All polymerase chain reactions (PCR) were conducted in a 30‐μL reaction volume, and began with initial denaturation at 94°C for 3 min, followed by 32 cycles of denaturation at 94°C for 40 s, annealing at 56°C for 60 s, extension at 72°C for 1 min, and a final extension at 72°C for 10 min. All PCR products were detected by 1% agarose gel electrophoresis. We then separated and purified the V4–V5 region of each product, and pooled an equal amount of the purified products for all samples to create sequencing libraries, which were sequenced using the Illumina MiSeq platform (Hangzhou, China). The raw 16S rRNA gene sequence data generated in this study have been deposited in the Genome Sequence Archive (https://www.cncb.ac.cn/) under accession number PRJCA040063.

### 2.4 Bioinformatics Analysis

We used the QIIME Pipeline (Yu et al. 2022) to handle the primitive 16S rRNA gene fastq sequence data, and used both FLASH 1.2.8 and Usearch 8.0 to eliminate low‐quality sequences (Yoon et al. 2017). Then, each read with more than 97% resemblance was aggregated into operational taxonomic units (OTUs) based on the UCLUST algorithm (Edgar 2010). Next, we used the “Daisy chopper” script (https://www.genomics.ceh.ac.uk/GeneSwytch/Tools.html) to level each OTU to reduce the influence of uneven sequencing (Gilbert et al. 2009). All samples were purified to the identical reads (11,168). Alpha diversity (Simpson index) was calculated based on the rarefied OTU abundance table. Beta diversity was estimated using Bray–Curtis dissimilarities and visualized using principal coordinate analysis (PCoA) (Yan et al. 2016). In this study, OTU clustering at 97% sequence similarity was used to maintain analytical consistency with previous long‐term monitoring studies of rodent gut microbiota in the same region. We acknowledge that ASV‐based approaches (e.g., DADA2) are now the current standard in microbial ecology, which can provide finer taxonomic resolution and better detection of rare taxa. The OTU‐based approach may slightly underestimate the diversity of rare taxa, and relevant conclusions should be interpreted with appropriate caution.

### 2.5 Statistical Analysis

We used one‐way analysis of variance (ANOVA) to analyze the differences in body size (carcass weight) and reproductive intensity (female pregnancy rate, male testicular descent rate) across monthly animal populations throughout 1 year, and the monthly variations of mean temperature, total precipitation and NDVI. Differences in alpha diversity between sexes were assessed using the Mann–Whitney *U* test. Monthly differences in alpha diversity were assessed using pairwise Mann–Whitney *U* tests. Differences in beta diversity were tested using permutational multivariate analysis of variance (PERMANOVA). If significant sex differences were detected, monthly microbial variation was analyzed separately for each sex; otherwise, samples were pooled for the monthly analysis. The community assembly process of gut bacteria in mice was quantified by the Modified Stochasticity Ratio (MST) model (Ning et al. 2019). The value of MST ranges from 0 to 1, with 0.5 serving as the threshold to determine whether community assembly is more deterministic (< 0.5) or more stochastic (> 0.5). We also used ANOVA to compare the difference in relative abundance for the top 10 phyla and genus in the gut bacterial community between reproductive and non‐reproductive period. For the significantly different phyla, the relative abundances of the class between the two periods were compared, and a similar comparison was extended sequentially to the order, family and genus levels.

We used the “psych” package (Revelle 2020) to analyze the pairwise correlations among the relative abundances of the top 200 operational taxonomic units (OTUs), and the co‐occurrence networks were visualized in Gephi 0.9.2 (Bastian et al. 2009) for the pairwise bacteria with a significant correlation (*p* < 0.05) and the absolute correlation coefficient was greater than 0.6. We quantified the topological properties of gut bacterial co‐occurrence networks through calculating standard metrics, including the number of nodes and edges, ratio of negative to positive sides, average degree, density, diameter, modularity, average clustering coefficient, and average path length.

We conducted redundancy analysis in the OmicStudio platform (https://www.OmicStudio.cn/tool) to evaluate the associations of environmental factors with gut bacterial community diversity, reproductive intensity and body size in *A. agrarius* . We constructed structural equation models (SEMs) as exploratory path analyses to examine associations among monthly environmental factors, gut bacterial alpha diversity, reproductive intensity, and body size. Individuals sampled within the same month shared the same environmental and population‐level reproductive predictor values; therefore, the SEMs were interpreted as exploratory association models rather than as tests of causal or mediating relationships. Because the effective replication of these month‐level predictors is limited, the strength of inference from the SEMs is constrained. Candidate models were compared using the Akaike Information Criterion (AIC). For each sex, the model with the lowest AIC was used as a representative exploratory model. Because several alternative models showed similar AIC values, the selected models were not interpreted as uniquely supported path structures (Bozdogan 1987). We conducted all analyses in R 3.6.3 (R Core Team 2020). The remaining figures were visualized via Origin 2021 (Northampton, USA).

## 3 Results

### 3.1 Monthly Variation of Reproductive Intensity and Body Size in *A. agrarius*

**FIGURE 1:**
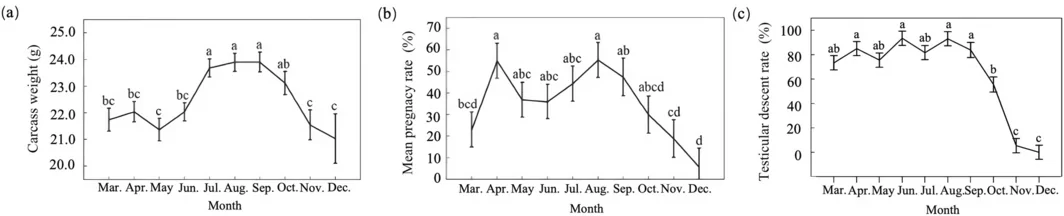
Monthly variations of (a) carcass weight, (b) mean pregnancy rate and (c) testicular descent rate in *A. agrarius* population. Error bars indicate standard errors, and lowercase letters above bars denote the significance of differences among months. Identical letters indicate no significant difference, and different letters indicate a significant difference.

*A. agrarius* population exhibited significant monthly variations in body size and reproductive intensity from March to December over 8 years. Carcass weight was the lowest in May (21.37 ± 0.43 g) and December (21.03 ± 0.93 g), and the highest weight was observed in August and September (23.90 ± 0.34 g and 23.91 ± 0.37 g) (Figure 1a). Female mean pregnancy rate displayed a bimodal pattern (Figure 1b), peaking in April (55.0% ± 8.1%) and August (55.4% ± 8.1%), and reaching the lowest value in December (5.7% ± 8.7%). The male testicular descent rate remained high from March to September (93.5% ± 5.8%), and declined to 0% ± 5.8% in December (Figure 1c).

### 3.2 Monthly Variation of Gut Bacterial Alpha Diversity in *A. agrarius*

**FIGURE 2:**
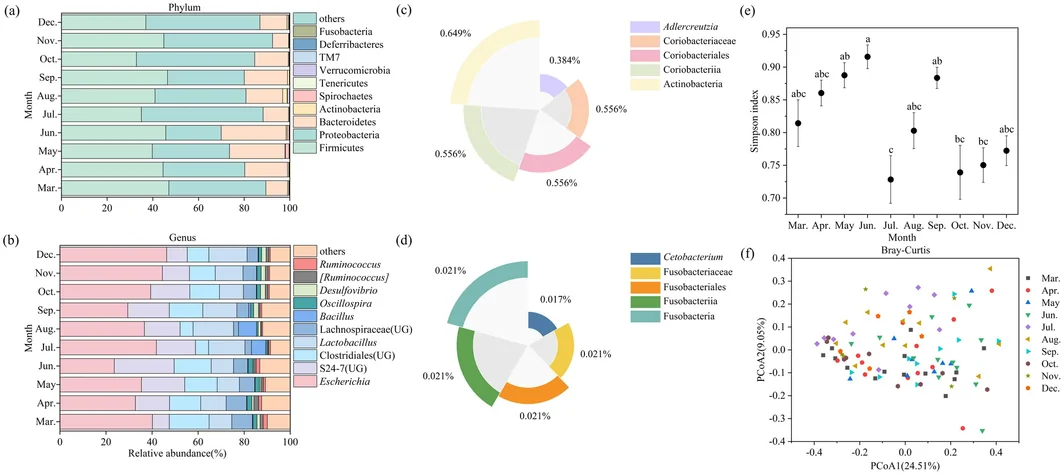
Monthly variations of gut bacterial community composition in *A. agrarius* populations at the (a) phylum and (b) genus level; relative abundances of bacterial taxa with significant differences between reproductive and non‐reproductive periods across multiple taxonomic levels within the (c) Actinobacteria and (d) Fusobacteria; the monthly variation of Alpha diversity for gut bacterial community (e), and the result of principal coordinate analysis (PCoA) for beta diversity in the community (f), where the *x*‐ and *y*‐axes represent the first and second principal coordinates, respectively, with percentages indicating the contribution of each component to sample variation.

As no significant differences were found in the gut bacterial community composition between males and females in *A. agrarius* (Figure S1), all samples were combined for the analysis of monthly variation, and significant differences were detected. Among the top 10 phyla (Figure 2a), Actinobacteria (0.649%) and Fusobacteria (0.021%) showed significantly higher relative abundance in the reproductive period than that in the non‐reproductive period, and other phyla remained stable between reproductive and non‐reproductive periods (Table S1). A total of six classes were identified in Actinobacteria and Fusobacteria, and the relative abundance of Coriobacteriia (0.556%) and Fusobacteriia (0.021%) in the two phyla was significantly higher in the reproductive period than in the non‐reproductive period of the animal (Table S2). Similar patterns existed in the corresponding orders (Coriobacteriales and Fusobacteriales) and families (Coriobacteriaceae and Fusobacteriaceae) (Tables S3 and S4). Finally, at the genus level, a total of nine genera were identified in Coriobacteriaceae and Fusobacteriaceae (Table S5), and only *Adlercreutzia* and *Cetobacterium* were significantly more abundant in the reproductive period than that in the non‐reproductive period, although the abundance of the two genera accounted for the smallest proportion of all genera in the gut microbiota (Figure 2b). The abundance of gut bacteria at different taxonomic categories in Actinobacteria and Fusobacteria was shown in Figure 2c,d.

Alpha diversity of the gut bacterial communities in the *A. agrarius* populations showed significant monthly variation from March to December (*p* < 0.05) as follows: the diversity value steadily increased from March to June, then dipped to a minimum in July, and rose again to a second peak in September; next, it decreased to its lowest point in October and again increased through November and December (Figure 2e). However, permutational multivariate analysis of variance (PERMANOVA) based on Bray–Curtis distance showed no significant seasonal difference in gut bacterial beta diversity (*p* > 0.05), and PCoA ordination revealed no distinct monthly clustering of community composition (Figure 2f). This indicates that seasonal variation is mainly reflected in alpha diversity, rather than substantial turnover of community composition.

**FIGURE 3:**
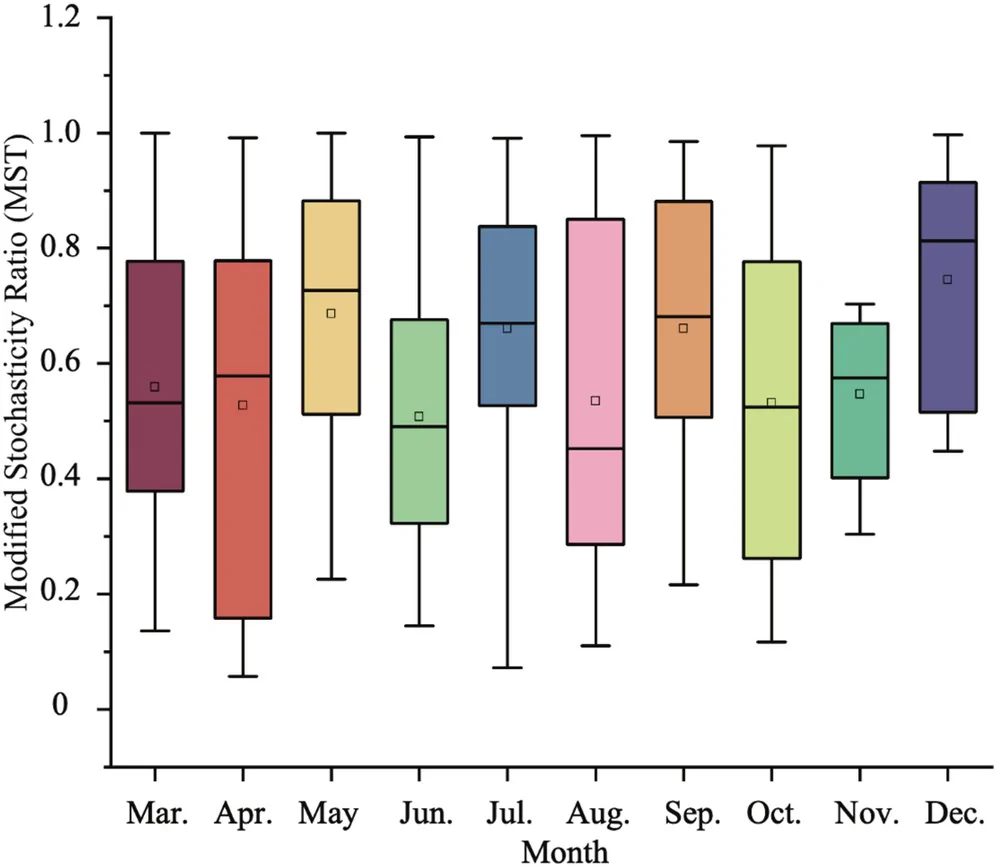
Monthly variation in the assembly mechanisms of gut bacterial communities across *A. agrarius* populations.

The results of Modified Stochasticity Ratio (MST) showed that the deterministic processes dominated the community assembly process of gut bacteria in June and August (MST < 0.5), while stochastic processes prevailed in other months (MST > 0.5) (Figure 3).

### 3.3 Monthly Co‐Occurrence Network Patterns of Bacterial Communities in *A. agrarius*

**FIGURE 4:**
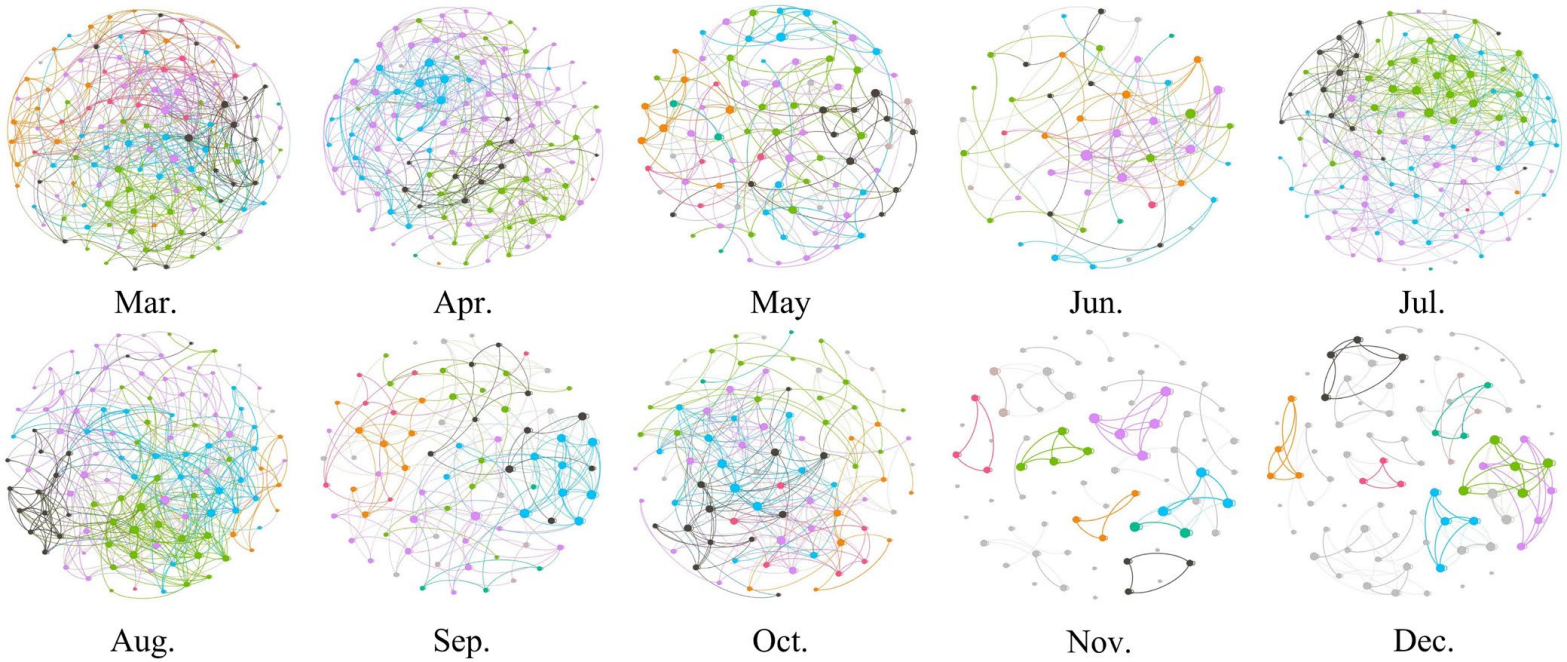
Monthly variation in gut bacterial co‐occurrence networks in *A. agrarius* populations. Nodes represent bacterial taxa, and edges represent significant co‐occurrence relationships. Modules indicate groups of bacterial taxa with relatively dense within‐module co‐occurrence relationships.

The dense and complex co‐occurrence network of gut bacterial communities in the monthly *A. agrarius* populations varied from March to December (Figure 4). During the reproductive periods (March–May and July–September), the co‐occurrence networks generally showed more nodes and edges than during the non‐reproductive periods, although patterns varied among individual network metrics. In the reproductive periods, the negative‐to‐positive edge ratio and average path length were generally higher, whereas the average clustering coefficient was lower than in the non‐reproductive periods. Complete monthly network topology parameters among bacterial taxa were presented in Table S6.

### 3.4 Relationships Among Environmental Factors, Reproductive Intensity, Growth Metrics and Gut Bacterial Community Structure in *A. agrarius*

**FIGURE 5:**
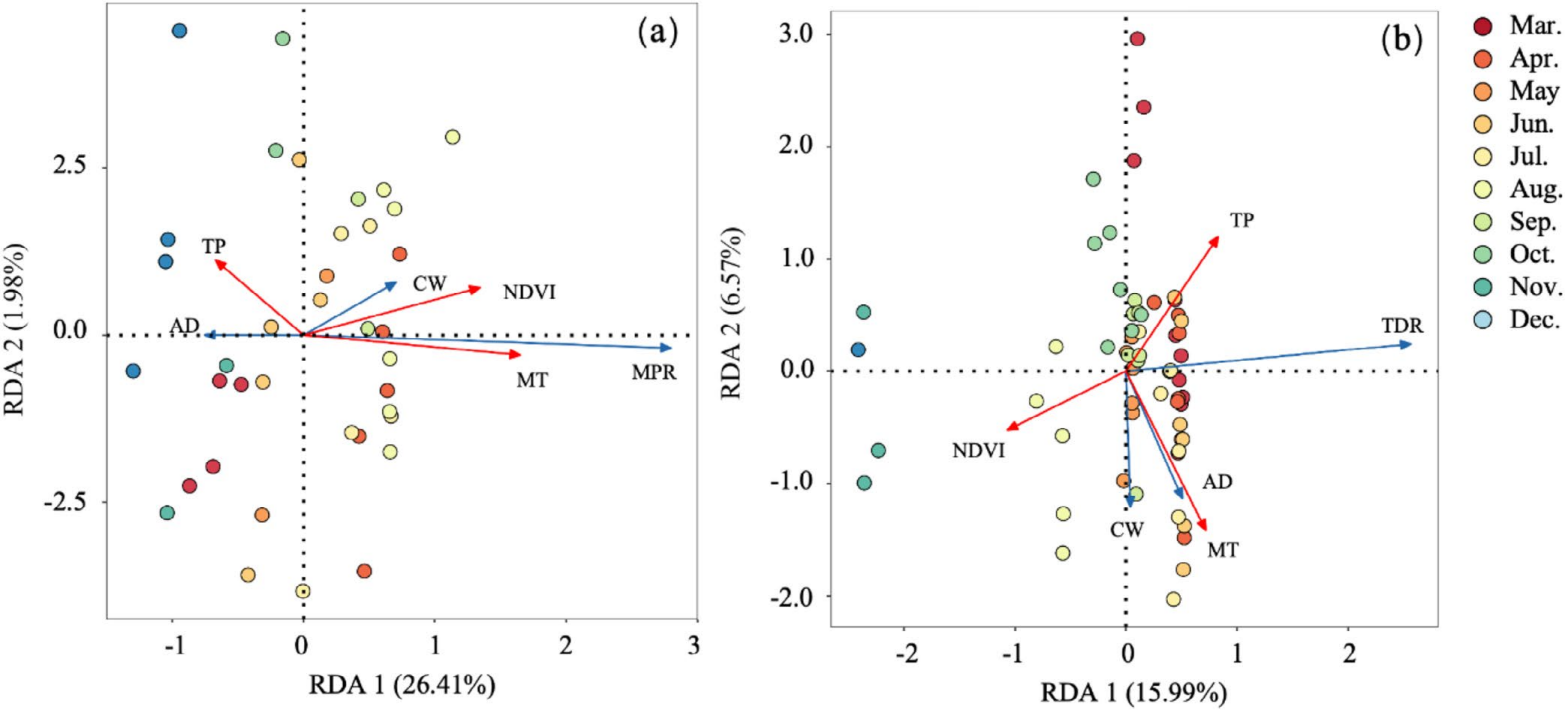
Results of redundancy analysis (RDA) showing associations of monthly mean temperature (MT), total precipitation (TP), and normalized difference vegetation index (NDVI) with the Simpson index of alpha diversity (AD) in the gut bacterial community, carcass weight (CW), and the monthly (a) mean pregnancy rate (MPR) or (b) testicular descent rate (TDR) in *A. agrarius* . The length of each factor arrow indicates the strength of the association with the corresponding response variables.

Mean temperature and NDVI showed significant monthly variation (*p* < 0.05), whereas total precipitation did not differ significantly among months (*p* > 0.05) (Figure S2). Redundancy analysis (RDA) revealed the significant associations between MT, TP and NDVI with gut microbial α‐diversity (AD), carcass weight (CW), and reproductive indices (Figure 5); in both sexes, CW was positively associated with NDVI, whereas reproductive indices (female mean pregnancy rate, MPR; male testis descent rate, TDR) increased with higher MT; and some sex‐specific relationships were found, in females, AD increased with more TP but decreased with higher MT and NDVI; MPR increased with higher NDVI but slightly declined with more TP (Figure 5a); in males, AD increased with higher MT and declined with more TP, showing no relationship with NDVI; male CW increased with higher MT but decreased with more TP, while TDR increased with more TP and decreased with higher NDVI (Figure 5b).

**FIGURE 6:**
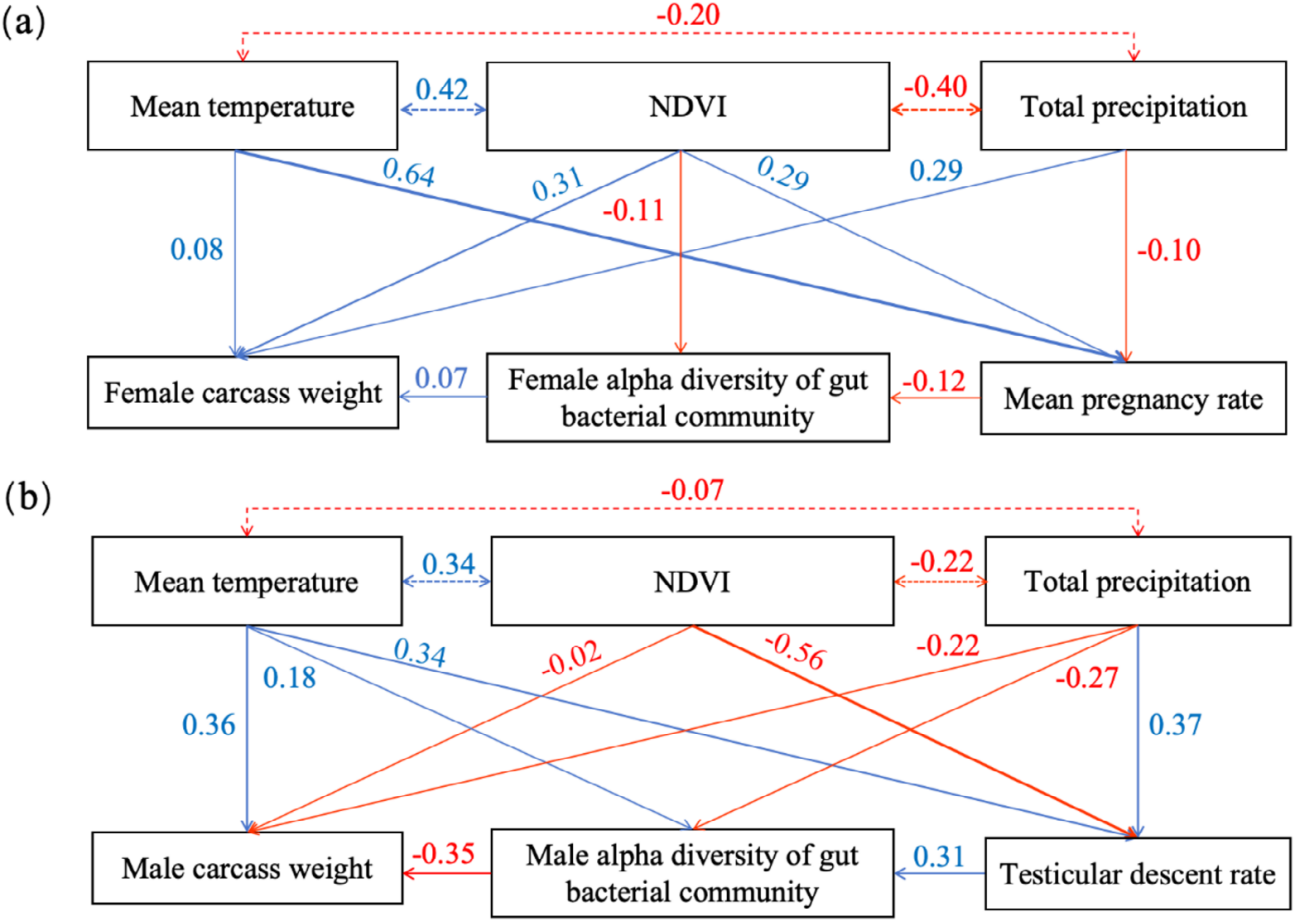
Structural equation models showing associations among monthly mean temperature (MT), total precipitation (TP), normalized difference vegetation index (NDVI), carcass weight, reproductive traits, and gut microbial alpha diversity in *A. agrarius* . (a) Model for females. (b) Model for males. Arrows indicate modeled paths. Solid and dashed lines represent significant (*p* < 0.05) and non‐significant (*p* > 0.05) paths, respectively. Arrow thickness is scaled to the absolute magnitude of the standardized estimates shown beside the arrows.

A total of 26 structural equation models across two categories were constructed as exploratory path analyses to examine association patterns among environmental variables, reproductive intensity, body size, and gut bacterial alpha diversity in *A. agrarius* (Tables S7 and S8). All 26 candidate models and their full fit statistics are provided in Tables S7 and S8. Among the candidate models, Model 8 for females (AIC = 89.734) and Model 3 for males (AIC = 237.444) had the lowest AIC values and were therefore used as representative exploratory models. Because several alternative models showed similar AIC values, these models were not considered uniquely supported. The selected exploratory models showed the following associations: In females, MPR was negatively associated with α‐diversity, which in turn was positively associated with CW. In males, TDR was positively associated with α‐diversity, which in turn was negatively associated with CW. Both models described sex‐specific association patterns among reproductive intensity, gut microbial α‐diversity, and carcass weight. These exploratory findings suggest potential sex‐specific correlational patterns that warrant further hypothesis‐driven investigation (Figure 6a,b).

## 4 Discussion

Our results demonstrated that the index of gut bacterial community structure in *A. agrarius* varied monthly, and monthly environmental factors, including mean temperature, total precipitation, and NDVI, were significantly associated with gut bacterial alpha diversity in the animal. The results also showed that reproductive status was associated with gut bacterial alpha diversity and carcass weight, with different association patterns between females and males in the animal. Together, these findings indicate that seasonal variation in gut bacterial community characteristics is associated with both external environmental factors and intrinsic reproductive status (Alberdi et al. 2016; Lochmiller and Deerenberg 2000).

### 4.1 Associations of Environmental Factors With the Gut Bacterial Community

Numerous studies have identified the effects of temperature, precipitation, and vegetation cover on the structure and function of gut microbiota in animal (Sepulveda and Moeller 2020; Fan et al. 2022). The present study found that increases in mean temperature and NDVI were associated with higher complexity of gut bacterial networks (Figure 4). One possible interpretation, based on previous studies, is that higher temperatures may be associated with changes in gut microbes involved in carbohydrate metabolism (Jiang et al. 2021). Furthermore, in the summer months, the vegetation is generally more abundant during summer, coinciding with higher NDVI values (Pettorelli et al. 2005), and animals have been observed to ingest greater quantities of plants with high fructose and oligofructose content (Li, Yin, et al. 2020); this dietary shift coincides with altered co‐occurrence network topology and a higher clustering coefficient, possibly reflecting shifts in interactions among fructose‐associated microbes (Li, Yin, et al. 2020). Conversely, increased NDVI may be associated with a higher abundance of plants containing high concentrations of secondary metabolites (e.g., high‐tannin plants). When animals consume such plants, the polyphenolic metabolites have been shown to bind to proteins on the outer membrane of some bacteria (e.g., proteolytic and fibrolytic bacteria), which may suppress bacterial growth (Besharati et al. 2022). This process may subsequently be linked to reduced gut microbiota diversity (Figure 6a). The observed associations between external environmental factors and gut microbial network structure suggest that environmental variation is correlated with shifts in microbial community organization, which may in turn be related to host growth patterns.

During the non‐reproductive months (June, November–February) (Figure 1b) (Zhang et al. 2025). Carcass weight was relatively high in June (Figure 1a), coinciding with relatively high temperature and NDVI and with monthly variation in gut bacterial alpha diversity (Figure 2e; Figure S2). From November to February, despite the lack of gut bacterial community data for January–February, results from November–December data indicate that reduced vegetation diversity and richness coincided with changes in microbial network (Figure 4) complexity and clustering coefficient (Table S6). Similar microbial network patterns have been associated with differences in microbial metabolic function in previous studies; however, microbial metabolic efficiency and host energy acquisition (Wolff et al. 2017) were not directly measured in the present study.

During the two reproductive periods of *A. agrarius* —spring (March–May) and summer–autumn (July–September)—gut microbial networks showed relatively high complexity and generally lower clustering coefficients (Figure 4; Table S6), along with a gradual increase in alpha diversity (Figure 2e). These microbial patterns coincided with periods of elevated reproductive activity. Animals in the reproductive period require more energy; the biological significance of the observed variation in alpha diversity during reproductive periods remains to be determined. Previous studies have proposed several possible functional implications of similar network structures; however, these interpretations remain speculative and were not directly tested in the present study, which only demonstrates structural correlations. It has been suggested that such network structures may be associated with various functional outcomes, including reduced diffusion loss of metabolic intermediates (Zengler and Zaramela 2018; D'Souza et al. 2018), directly supplying energy to the host (Den Besten et al. 2013), inhibiting nutrient degradation by potentially harmful microorganisms (Kamada et al. 2013; Sassone‐Corsi and Raffatellu 2015), improving metabolite transport efficiency (Martens et al. 2018), and decreasing energy expenditure for nutrient absorption and body maintenance (Tremaroli and Bäckhed 2012; Nieuwdorp et al. 2014). However, these functional interpretations remain speculative and are not directly tested in the present study, which only demonstrates structural correlations. Similarly, the reduction in the clustering coefficient of the microbial co‐occurrence network has been hypothesized to reflect decreased competition among different microbial taxa (Peura et al. 2015), which could be associated with enhanced food digestion, synthesis of key nutrients, and production of reproduction‐related bioactive compounds (Zhang et al. 2023). Whether such structural changes indeed correspond to the increased energy and material requirements of animal reproduction (Lombardo 2008) requires further functional validation. However, the observed seasonal patterns in gut microbial structure cannot be attributed solely to seasonal variation in environmental factors. For instance, temperatures are low and vegetation cover is sparse in the first reproductive period (spring), whereas both of them are higher in the second peak period (summer–autumn); yet, gut microbial structures share consistent features in the two reproductive periods. The similarity of microbial patterns across reproductive periods suggests that reproductive status may also be associated with seasonal microbial variation. Because reproductive hormones were not measured, the contribution of hormonal changes cannot be determined from the present study.

### 4.2 Associations of Intrinsic Physiological Status With the Gut Bacterial Community

Reproductive hormone fluctuations have been associated with gut microbial variation in mammals. Therefore, hormone‐related explanations should be regarded as hypotheses rather than mechanisms demonstrated by our data. Although we did not measure hormonal levels in this study, we speculate that the observed microbial changes during the reproductive period may be related to seasonal shifts in reproductive hormones such as estrogen and testosterone, as well as HPG axis regulation.

Previous studies have reported associations between gut microbiota and gonadal steroid profiles (Mayneris‐Perxachs et al. 2020). Although there is no direct evidence supporting the influence of hormones on the gut microbial community structure in our results, there are numerous reports on the dramatic fluctuations in reproductive‐related hormones during animal reproductive periods (Bigler et al. 2023). Our exploratory SEMs identified sex‐specific and opposite associations among reproductive status, gut microbial alpha diversity, and carcass weight. At the taxonomic level, gut bacterial composition differed between reproductive and non‐reproductive periods. In our results, Actinobacteria and Fusobacteria were significantly enriched (Table S1), and the relative abundance of reproduction‐associated genera such as *Adlercreutzia* and *Cetobacterium* (Mayneris‐Perxachs et al. 2020; Garrett and Uno 2021) increased (Table S5). Previous studies have shown that some *Adlercreutzia* strains can metabolize soy isoflavones to equol; however, whether this function occurs in *A. agrarius* or contributes to reproduction was not examined in the present study (Vázquez et al. 2020). For example, synergistic metabolic functions of amino acid synthesis and short‐chain fatty acid production were enhanced by the specific gut microbiota (Zhang et al. 2023).

Variation in gut microbial structure associated with reproductive status in *A. agrarius* was also related to host carcass weight, with opposite patterns observed between females and males. In the selected exploratory SEMs, reproductive intensity was associated with gut microbial alpha diversity, with contrasting patterns between females and males (Figure 6). In females, higher MPR was associated with reduced alpha diversity and higher CW. Pregnancy‐associated changes in gut microbiota and host metabolism reported in other mammals may provide one possible context for this pattern; however, these mechanisms were not directly examined in the present study (Koren et al. 2012; Nuriel‐Ohayon et al. 2016). In males, higher TDR was associated with increased alpha diversity but lower CW. The physiological basis of this association remains unresolved (Zhu et al. 2022). Variation in alpha diversity during reproductive periods coincided with enrichment of Adlercreutzia and Cetobacterium (Figure 2a,b; Table S5). However, these genera occurred at very low relative abundances, and their ecological and physiological significance remains uncertain. Reported functions of these genera in other hosts may provide hypotheses for future investigation; however, their physiological roles in *A. agrarius* remain unknown. Their actual physiological roles in this species remain to be verified by functional studies (Maurice et al. 2015). Notably, these differentially enriched genera (*Adlercreutzia* and *Cetobacterium*) occur at very low relative abundances in the gut microbiota of *A. agrarius* , and their ecological significance remains to be further evaluated. The opposite association patterns between sexes indicate prominent sexual dimorphism in the gut microbiota–phenotype relationship of *A. agrarius* . The opposite association patterns observed between females and males indicate sex‐specific relationships among reproductive status, gut microbial diversity, and carcass weight. The underlying physiological mechanisms remain unresolved and require experimental validation.

## 5 Conclusions

An important limitation of this study is the temporal mismatch between the ecological and microbiome datasets. Host phenotypic and reproductive data were collected from 2016 to 2023, whereas gut microbiota were sampled only in 2023. Therefore, the microbiome data should be interpreted as a single‐year seasonal snapshot within the context of longer‐term ecological patterns rather than as evidence explaining interannual variation across the eight‐year period.

Our results revealed a correlated variation pattern among environmental factors, gut microbial alpha diversity, and host phenotypes in *A. agrarius* . Seasonal environmental variation was associated with gut microbial alpha diversity and reproductive intensity, while reproductive intensity was also associated with microbial alpha diversity in the exploratory SEMs. Together, these findings suggest that gut microbial alpha diversity covaries with seasonal environmental conditions and host life‐history traits. The mechanisms underlying these associations remain hypotheses requiring experimental validation. Whether these associations contribute to seasonal adaptation requires further experimental and longitudinal investigation.

## Author Contributions

**Yizhen Zhang:** conceptualization (lead), data curation (lead), formal analysis (lead), investigation (lead), methodology (lead), software (lead), visualization (lead), writing – original draft (lead), writing – review and editing (equal). **Yan Chen:** conceptualization (equal), data curation (equal), software (supporting), visualization (supporting). **Zhijiang Bai:** conceptualization (supporting), data curation (equal). **Xinliang Zhang:** writing – review and editing (equal). **Deng Wang:** conceptualization (equal), funding acquisition (lead), methodology (supporting), project administration (lead), resources (lead), supervision (equal), writing – review and editing (lead).

## Funding

This study was funded by the National Key Research and Development Program of China (grant no. 2024YFD1400500) and the National Natural Science Foundation of China (grant no. 32272561).

## Conflicts of Interest

The authors declare no conflicts of interest.

## Supporting information


**Figure S1:** Sex differences in gut microbial (a) alpha diversity (Simpson index) and (b) beta diversity in *A. agrarius* sampled from March to December 2023.
**Figure S2:** Monthly variations in (a) mean temperature, (b) total precipitation, and (c) Normalized Difference Vegetation Index (NDVI) at the sampling sites of *A. agrarius* population. The boxes in the figures represent the mean values, and the horizontal lines inside the boxes represent the median values; different lowercase letters marked above the box—plots indicate significant differences between groups (*p* < 0.05).
**Table S1:** Differences of top 10 gut bacterial phyla in *A. agrarius* population between non‐reproductive and reproductive periods.
**Table S2:** Differences of the class belonging to Actinobacteria and Fusobacteria in *A. agrarius* population between non‐reproductive and reproductive periods.
**Table S3:** Differences of Coriobacteriales and Fusobacteriales in *A. agrarius* population between non‐reproductive and reproductive periods.
**Table S4:** Differences of Coriobacteriaceae and Fusobacteriaceae in *A. agrarius* population between non‐reproductive and reproductive periods.
**Table S5:** Differences of the nine genera belonging to Coriobacteriaceae and Fusobacteriaceae in *A. agrarius* population between non‐reproductive and reproductive periods.
**Table S6:** Monthly variations for the network topology parameters of gut bacteria in *A. agrarius* populations.
**Table S7:** Structural Equation Model illustrating the relationships among mean temperature, total precipitation, NDVI, the Alpha diversity of the gut bacterial community, carcass weight and mean pregnancy rate in female *A. agrarius* populations. Rectangles of different colors denote the variables as follows: blue for Mean temperature; orange for NDVI; green for total precipitation; pink for female carcass weight; yellow for the Alpha diversity (Simpson index) of gut bacterial community in female; gray for mean pregnancy rate.
**Table S8:** Structural equation model illustrating the relationships among the Alpha diversity of the gut bacterial community, carcass weight and testicular descent rate in male A. agrarius populations, and mean temperature, total precipitation, NDVI. Rectangles of different colors denote the variables as follows: light pink for male carcass weight; light yellow for the Alpha diversity (Simpson index) of gut bacterial community in male; brown for testicular descent rate; others are the same as in Table S7.

## Data Availability

The data that support the findings of this study are openly available in the Genome Sequence Archive (GSA) at https://ngdc.cncb.ac.cn/gsa/, under accession number PRJCA040063. The remaining data and code are available in the Supporting Information.
