# Supplementary material for: Seasonal Variation of Gut Bacterial Community in Wild Apodemus agrarius Is Associated With Intrinsic and Extrinsic Environments
Source: Ecol Evol. 2026 Sep 1;16(9):e74257. doi: 10.1002/ece3.74257 (PMC13531371; doi:10.1002/ece3.74257)
Supplement: Supplementary file 1 [file ECE3-16-e74257-s001.doc]

**Electronic supplementary material**

**Seasonal variation of gut bacterial community in wild *Apodemus agrarius* is associated with intrinsic and extrinsic environments**

**List of figures and tables in the supporting attachment**

| Item | Content |
| --- | --- |
| Figure S1 | Sex differences in gut microbial (a) alpha diversity (Simpson index) and (b) beta diversity in *A. agrarius* sampled from March to December 2023. |
| Table S1 | Differences of top 10 gut bacterial phyla in *A. agrarius* population between non-reproductive and reproductive periods |
| Table S2 | Differences of the class belonging to Actinobacteria and Fusobacteria in *A. agrarius* population between non-reproductive and reproductive periods |
| Table S3 | Differences of Coriobacteriales and Fusobacteriales in *A. agrarius* population between non-reproductive and reproductive periods |
| Table S4 | Differences of Coriobacteriaceae and Fusobacteriaceae in *A. agrarius* population between non-reproductive and reproductive periods |
| Table S5 | Differences of the nine genera belonging to Coriobacteriaceae and Fusobacteriaceae in *A. agrarius* population between non-reproductive and reproductive periods |
| Table S6 | Monthly variations for the network topology parameters of gut bacteria in *A.agrarius* populations |
| Figure S2 | Monthly variations in (a) mean temperature, (b) total precipitation, and (c) Normalized Difference Vegetation Index (NDVI) at the sampling sites of *A. agrarius* population |
| Table S7 | Structural Equation Model illustrating the relationships among mean temperature, total precipitation, NDVI, the female Alpha diversity of the gut bacterial community, female carcass weight and mean pregnancy rate in *A. agrarius* populations |
| Table S8 | Structural equation model illustrating the relationships among the male Alpha diversity of the gut bacterial community, male carcass weight and testicular descent rate in *A. agrarius* populations, and mean temperature, total precipitation, NDVI |

**Figure S1** Sex differences in gut microbial (a) alpha diversity (Simpson index) and (b) beta diversity in *A. agrarius* sampled from March to December 2023.

**Table S1** Differences of top 10 gut bacterial phyla in *A. agrarius* population between non-reproductive and reproductive periods

| phyla | *F* | *p* |
| --- | --- | --- |
| Firmicutes | 0.585 | 0.446 |
| Proteobacteria | 1.290 | 0.259 |
| Bacteroidetes | 0.390 | 0.534 |
| Actinobacteria | 6.361 | 0.013^*^ |
| Spirochaetes | 1.715 | 0.194 |
| Tenericutes | 0.228 | 0.634 |
| Verrucomicrobia | 1.764 | 0.187 |
| TM7 | 2.528 | 0.115 |
| Deferribacteres | 1.834 | 0.179 |
| Fusobacteria | 5.468 | 0.021^*^ |

**Note:** * indicates *p* < 0.05, and the same applies below.

**Table S2** Differences of the class belonging to Actinobacteria and Fusobacteria in *A. agrarius* population between non-reproductive and reproductive periods

| class | *F* | *p* |
| --- | --- | --- |
| Acidimicrobiia | 2.370 | 0.127 |
| Actinobacteria | 3.852 | 0.053 |
| Coriobacteriia | 5.083 | 0.026^*^ |
| Rubrobacteria | 1.011 | 0.317 |
| Thermoleophilia | 0.625 | 0.431 |
| Fusobacteriia | 5.468 | 0.021^*^ |

**Table S3** Differences of Coriobacteriales and Fusobacteriales in *A. agrarius* population between non-reproductive and reproductive periods

| order | *F* | *p* |
| --- | --- | --- |
| Coriobacteriales | 5.083 | 0.026^*^ |
| Fusobacteriales | 5.468 | 0.021^*^ |

**Table S4** Differences of Coriobacteriaceae and Fusobacteriaceae in *A. agrarius* population between non-reproductive and reproductive periods

| family | *F* | *p* |
| --- | --- | --- |
| Coriobacteriaceae | 5.083 | 0.026^*^ |
| Fusobacteriaceae | 5.468 | 0.021^*^ |

**Table S5** Differences of the nine genera belonging to Coriobacteriaceae and Fusobacteriaceae in *A. agrarius* population between non-reproductive and reproductive periods

| genera | *F* | *p* |
| --- | --- | --- |
| Coriobacteriaceae(UG) | 0.033 | 0.857 |
| *Adlercreutzia* | 5.525 | 0.021^*^ |
| *Atopobium* | 0.000 | 1.000 |
| *Collinsella* | 0.000 | 1.000 |
| *Coriobacterium* | 2.288 | 0.134 |
| *Slackia* | 0.000 | 1.000 |
| *Cetobacterium* | 5.373 | 0.023^*^ |
| *Fusobacterium* | 1.001 | 0.320 |
| *u114* | 2.022 | 0.158 |

**Table S6** Monthly variations for the network topology parameters of gut bacteria in *A.agrarius* populations

|  | Mar. | Apr. | May | Jun. | Jul. | Aug. | Sep. | Oct. | Nov. | Dec. |
| --- | --- | --- | --- | --- | --- | --- | --- | --- | --- | --- |
| Node | 124 | 112 | 86 | 50 | 101 | 114 | 81 | 86 | 73 | 83 |
| Edge | 653 | 538 | 294 | 157 | 533 | 600 | 280 | 357 | 134 | 197 |
| Negative side：Positive side | 0.29 | 0.46 | 0.41 | 0.20 | 0.30 | 0.32 | 0.35 | 0.25 | 0.19 | 0.32 |
| Average degree | 10.53 | 9.61 | 6.84 | 6.28 | 10.55 | 10.53 | 6.91 | 8.30 | 3.67 | 4.75 |
| Diameter | 5 | 6 | 8 | 9 | 7 | 7 | 10 | 8 | 1 | 1 |
| Density | 0.09 | 0.09 | 0.08 | 0.13 | 0.11 | 0.09 | 0.09 | 0.10 | 0.05 | 0.06 |
| Modularity | 0.92 | 1.98 | 2.71 | 0.69 | 0.95 | 1.11 | 1.66 | 0.89 | 1.61 | 2.89 |
| Average clustering coefficient | 0.46 | 0.54 | 0.56 | 0.62 | 0.57 | 0.56 | 0.61 | 0.60 | 0.93 | 0.96 |
| Average path length | 2.74 | 2.99 | 3.77 | 3.84 | 2.82 | 3.02 | 3.87 | 3.20 | 1.00 | 1.00 |

**Figure S2** Monthly variations in (a) mean temperature, (b) total precipitation, and (c) Normalized Difference Vegetation Index (NDVI) at the sampling sites of *A. agrarius* population. The boxes in the figures represent the mean values, and the horizontal lines inside the boxes represent the median values; different lowercase letters marked above the box - plots indicate significant differences between groups (*p* < 0.05).

**Table S7** Structural Equation Model illustrating the relationships among mean temperature, total precipitation, NDVI, the Alpha diversity of the gut bacterial community, carcass weight and mean pregnancy rate in female *A. agrarius* populations. Rectangles of different colors denote the variables as follows: blue for Mean temperature; orange for NDVI; green for total precipitation; pink for female carcass weight; yellow for the Alpha diversity (Simpson index) of gut bacterial community in female; gray for mean pregnancy rate.

| Model | Structure | χ^2^ | d.f | *p*-value | CFI | GFI | RMSEA | SRMR | AIC |
| --- | --- | --- | --- | --- | --- | --- | --- | --- | --- |
| Model 1 |  | 0.000 | 0.000 | NA | 1.000 | 1.000 | 0.000 | 0.000 | 95.306 |
| Model 2 |  | 0.684 | 1.000 | 0.408 | 1.000 | 0.996 | 0.000 | 0.031 | 93.990 |
| Model 3 |  | 0.312 | 1.000 | 0.576 | 1.000 | 0.998 | 0.000 | 0.018 | 93.619 |
| Model 4 |  | 0.027 | 1.000 | 0.870 | 1.000 | 1.000 | 0.000 | 0.005 | 93.333 |
| Model 5 |  | 0.719 | 2.000 | 0.698 | 1.000 | 0.996 | 0.000 | 0.032 | 92.025 |
| Model 6 |  | 1.566 | 2.000 | 0.457 | 1.000 | 0.991 | 0.000 | 0.061 | 92.873 |
| Model 7 |  | 0.783 | 3.000 | 0.854 | 1.000 | 0.995 | 0.000 | 0.033 | 90.089 |
| Model 8 |  | 0.453 | 3.000 | 0.929 | 1.000 | 0.997 | 0.000 | 0.014 | 89.734 |
| Model 9 |  | 0.453 | 3.000 | 0.929 | 1.000 | 0.997 | 0.000 | 0.014 | 89.759 |
| Model 10 |  | 0.719 | 2.000 | 0.698 | 1.000 | 0.996 | 0.000 | 0.032 | 92.025 |
| Model 11 |  | 0.440 | 3.000 | 0.932 | 1.000 | 0.997 | 0.000 | 0.015 | 89.746 |
| Model 12 |  | 0.719 | 2.000 | 0.698 | 1.000 | 0.996 | 0.000 | 0.032 | 92.025 |
| Model 13 |  | 0.719 | 2.000 | 0.698 | 1.000 | 0.996 | 0.000 | 0.032 | 92.025 |
| Model 14 |  | 0.293 | 1.000 | 0.588 | 1.000 | 0.998 | 0.000 | 0.015 | 93.599 |
| Model 15 |  | 0.286 | 1.000 | 0.592 | 1.000 | 0.998 | 0.000 | 0.015 | 93.593 |

**Table S8** Structural equation model illustrating the relationships among the Alpha diversity of the gut bacterial community, carcass weight and testicular descent rate in male *A. agrarius* populations, and mean temperature, total precipitation, NDVI. Rectangles of different colors denote the variables as follows: light pink for male carcass weight; light yellow for the Alpha diversity (Simpson index) of gut bacterial community in male; brown for testicular descent rate; others are the same as in **Table S7**.

| Model | Structure | χ^2^ | d.f | p-value | CFI | GFI | RMSEA | SRMR | AIC |
| --- | --- | --- | --- | --- | --- | --- | --- | --- | --- |
| Model 1 |  | 1.105 | 1.000 | 0.293 | 0.998 | 0.991 | 0.042 | 0.033 | 239.056 |
| Model 2 |  | 2.483 | 2.000 | 0.289 | 0.991 | 0.979 | 0.064 | 0.036 | 238.434 |
| Model 3 |  | 1.493 | 2.000 | 0.474 | 1.000 | 0.987 | 0.000 | 0.022 | 237.444 |
| Model 4 |  | 3.189 | 2.000 | 0.203 | 0.977 | 0.974 | 0.100 | 0.037 | 239.140 |
| Model 5 |  | 1.105 | 1.000 | 0.293 | 0.998 | 0.991 | 0.042 | 0.033 | 239.056 |
| Model 6 |  | 3.186 | 2.000 | 0.203 | 0.977 | 0.974 | 0.100 | 0.038 | 239.137 |
| Model 7 |  | 1.105 | 1.000 | 0.293 | 0.998 | 0.991 | 0.042 | 0.033 | 239.056 |
| Model 8 |  | 1.105 | 1.000 | 0.293 | 0.998 | 0.991 | 0.042 | 0.033 | 239.056 |
| Model 9 |  | 0.007 | 1.000 | 0.933 | 1.000 | 1.000 | 0.000 | 0.002 | 237.958 |
| Model 10 |  | 0.099 | 1.000 | 0.753 | 1.000 | 0.999 | 0.000 | 0.007 | 238.050 |
| Model 11 |  | 0.099 | 1.000 | 0.753 | 1.000 | 0.999 | 0.000 | 0.007 | 238.050 |
